# Effects of the encapsulation of usnic acid into liposomes and interactions with antituberculous agents against multidrug-resistant tuberculosis clinical isolates

**DOI:** 10.1590/0074-02760150454

**Published:** 2016-05

**Authors:** Rafaela S Ferraz-Carvalho, Marcela A Pereira, Leonardo A Linhares, Mariane CB Lira-Nogueira, Isabella MF Cavalcanti, Nereide S Santos-Magalhães, Lílian ML Montenegro

**Affiliations:** 1 Universidade Federal de Pernambuco, Laboratório de Imunopatologia Keizo-Asami, Recife, PE, Brasil; 2 Fundação Oswaldo Cruz, Centro de Pesquisas Aggeu Magalhães, Departamento de Imunologia,Laboratório de Imunoepidemiologia, Recife, PE, Brasil; 3 Universidade Federal de Pernambuco, Centro Acadêmico de Vitória, Laboratório de Microbiologia e Imunologia, Vitória de Santo Antão, PE, Brasil

**Keywords:** MDR-TB, antimycobacterial activity, synergism, usnic acid, liposomes

## Abstract

*Mycobacterium tuberculosis* (Mtb) has acquired resistance and consequently the antibiotic therapeutic options available against this microorganism are limited. In this scenario, the use of usnic acid (UA), a natural compound, encapsulated into liposomes is proposed as a new approach in multidrug-resistant tuberculosis (MDR-TB) therapy. Thus the aim of this study was to evaluate the effect of the encapsulation of UA into liposomes, as well as its combination with antituberculous agents such as rifampicin (RIF) and isoniazid (INH) against MDR-TB clinical isolates. The in vitro antimycobacterial activity of UA-loaded liposomes (UA-Lipo) against MDR-TB was assessed by the microdilution method. The in vitro interaction of UA with antituberculous agents was carried out using checkerboard method. Minimal inhibitory concentration values were 31.25 and 0.98 µg/mL for UA and UA-Lipo, respectively. The results exhibited a synergistic interaction between RIF and UA [fractional inhibitory concentration index (FICI) = 0.31] or UA-Lipo (FICI = 0.28). Regarding INH, the combination of UA or UA-Lipo revealed no marked effect (FICI = 1.30-2.50). The UA-Lipo may be used as a dosage form to improve the antimycobacterial activity of RIF, a first-line drug for the treatment of infections caused by Mtb*.*

Tuberculosis (TB) is a chronic bacterial infection caused by an airborne microorganism known as *Mycobacterium tuberculosis* (Mtb). The treatment for susceptible Mtb isolates is based on a combination of isoniazid (INH), rifampicin (RIF), ethambutol (EMB) and pyrazinamide (PZA) (WHO 2013). Patients with TB infection usually require a long-term therapy and non-compliance with the full therapeutic regimen may lead to the emergence of multi- and extensively drug-resistant Mtb (MDR-TB and XDR-TB) strains (Ganihigama et al. 2015). In 2010, only 48% of MDR-TB patients worldwide were successfully treated with the currently used anti-TB drugs (WHO 2013). The therapeutic options for the treatment of MDR-TB are PZA concurrently with second-line drugs such as ethionamide, prothionamide, clycloserine, capreomycin, p-aminosalicylic acid or fluoroquinolones (Mukherjee et al. 2004). Since the second-line of antituberculous drugs exhibit more toxicity, are more expensive and less potent than the first-line agents (Kaur & Singh 2014), there is an urgent need to find new effective drugs, new forms of drug administration and combinations of antituberculous agents for the treatment of TB.

In the last few years, many new synthetic or natural agents have been tested against Mtb in order to discover compounds capable of replacing or complementing established drugs used in TB therapy (WHO 2013). Usnic acid (UA), a lichen dibenzofuran derivative, has been shown to have an interesting antimycobacterial activity (Ingólfsdóttir et al. 1998). However, its weak potency compared with reference antimycobacterial drugs does not allow its use as an antituberculous drug (Yempala et al. 2013).

Moreover, nanotechnology has emerged as an efficient tool able to enhance drug efficacy and overcome the resistance of *Mycobacterium* against well-known antibiotics usually prescribed in clinical practice (Yempala et al. 2013). These advantages, associated with the fact that only one anti-TB drug (TMC207) has been approved by the United States Food and Drug Administration in the last four decades, indicate the feasibility of nanosystems, such as liposomes containing antimycobacterial drugs (Ganihigama et al. 2015). Liposome encapsulation has long been shown to improve the therapeutic efficacy of antimicrobial drugs (El-Ridy et al. 2007, Gaspar et al. 2008). More recently, proliposomes containing levofloxacin have been used in the form of Mtb treatment with promising results (Rojanarat et al. 2012). With regard to the combination of drugs, pulmonary TB and further drug-resistant cases almost invariably require a combination of multidrug regimens over long periods of treatment (Pham et al. 2015). Therefore, the combination of first-line drugs (e.g. RIF or INH) with UA in drug delivery systems, emerges as an approach to take advantage in TB therapy (Bapela et al. 2006, Ganihigama et al. 2015).

Although the in vitro and in vivo antimicrobial activity of UA (Marshak & Kuschner 1950, Honda et al. 2010, Ramos & Silva 2010), and UA encapsulated into liposomes against Mtb is well established (Lira et al. 2009), their activity against MDR-TB, as well as their interactions with RIF and INH, has not yet been investigated. Thus, the aim of our study was to evaluate the effect of UA encapsulation into liposomes (UA-Lipo) and its combinations with RIF or INH, against MDR-TB clinical isolates.

## MATERIALS AND METHODS

INH, RIF, (+)-UA, cholesterol (Chol), stearylamine (SA) and Middlebrook 7H9 medium were obtained from Sigma-Aldrich (St Louis, MO, USA). Middlebrook OADC Enrichment was purchased from Becton Dickinson (New Jersey, USA). Soybean phosphatidylcholine (PC) (94% Epikuron 200^®^) was furnished by Lipoid GMBH (Ludwigshafen, Germany). All solvents and other chemicals were supplied by Merck (Darmstadt, Germany).

*Preparation and characterisation of liposomes containing UA* - Positively-charged liposomes containing UA-Lipo were prepared using lipids at 80 mM (soya PC, Chol and SA; 8:1:1) and UA (2 mg/mL) by the thin film method. Briefly, lipid constituents and UA were dissolved in a mixture of chloroform and methanol (3:1 v/v) under magnetic stirring. The solvents were removed under pressure (37ºC, 80 rpm) and the thin film formed was hydrated with 10 mL of phosphate buffer solution (7.4 pH). The liposomal dispersion was then sonicated (Vibra Cell, BRANSON, USA) at 200 W and 40 Hz for 300 s in order to obtain small unilamellar vesicles.

The mean particle size and polydispersity index of liposomes were measured by photon autocorrelation spectroscopy using a laser particle analyser Delsa TM Nano-S (Beckman Coulter, UK). The zeta potential of the liposomes was determined by electrophoretic mobility using a ZetasizerNano-ZS90 (Malvern, Worcestershire, UK). Analyses were performed using samples diluted with deionised water (2:1) at 25ºC. Moreover, the encapsulation efficiency of UA into liposomes was determined using ultrafiltration/centrifugation by a previously validated method (Lira et al. 2009).

*Antimycobacterial activity* - *Mycobacterium strains and growth conditions* - The Mtb H37Rv ATCC 27294 strain and six MDR-TB clinical isolates were studied (MDR-TB 1412, 1619, 0729, 1411, 1409 and 1484). MDR-TB isolates were obtained from the Central Public Health Laboratory of Pernambuco (LACEN/PE, Brazil). Drug susceptibility of MDR-TB isolates to first-line drugs [EMB, streptomycin (SM), INH and RIF] was verified using standard agar methods (LACEN, PE, Brazil). Mtb strains were cultured in Middlebrook 7H9 broth supplemented with 10% (v/v) OADC, 0.05% (v/v) Tween 80 (Sigma-Aldrich) and 0.2% (v/v) glycerol (Sigma-Aldrich) and incubated at 37°C for approximately 10 days.

*Determination of the minimum inhibitory concentration (MIC)* - The antimycobacterial activity of the tested samples (UA and UA-Lipo) and the reference drugs, INH and RIF, was determined in triplicate through the Microplate Alamar Blue Assay (CLSI 2011). Initially, all the 96-well microplates were filled with 100 µL of Middlebrook 7H9 broth. Serial two-fold dilutions of drugs were made by transferring 100 µL from the first to the last well. INH solution was prepared in sterile distilled water and diluted in 7H9 broth to a concentration from 16 to 0.25 µg/mL. RIF and UA were dissolved in 0.5% methanol (8-0.125 µg/mL and 125-1.95 µg/mL, respectively) and UA-Lipo was dissolved in Middlebrook 7H9 broth (500-0.122 µg/mL). Finally, the concentrations of H37Rv ATCC 27294 and MDR-TB clinical isolates were adjusted to a density corresponding to 1.0 McFarland turbidity standards, followed by dilution (1:20 v/v) in 7H9 broth. Each well was inoculated with 100 µL of this bacterial suspension. Plates were incubated at 37 ± 1ºC for approximately 10 days. Next, 30 µL of freshly prepared resazurin (0,01%) was added to each well and incubated for 24 h. Growth of the microorganisms after reincubation at 37 ± 1ºC for 24 h was verified by visual determination of a colour change from blue to pink. The MIC is defined as the lowest drug concentration that prevents the colour change. The positive and negative control wells were consisted of Middlebrook 7H9 broth with bacterial suspension, and Middlebrook 7H9 broth plus the tested drugs, respectively. Methanol (0.5%) and unloaded liposomes (40-0.625 mM total lipid concentration) were used to evaluate any possible effects of solvents and liposomal constituents on bacterial growth. All the experiments were performed in triplicate.

*Checkerboard method* - The in vitro interactions between RIF or INH and UA or UA-Lipo were evaluated by the checkerboard bidimensional method (Agertt et al. 2013). Initially, the 96-well microplates were seeded by dispersing 100 µL of Middlebrook 7H9 broth into each well. Next, it was dispensed in the X-axis of the 96-well microdilution plates the serially diluted standards (RIF or INH) and in the Y-axis the testing drugs (UA or UA-Lipo) to obtain a final concentration equal to the MIC or dilutions lower than the MIC of the respective drugs. Finally, the bacterial concentration was adjusted to a density corresponding to 1.0 McFarland turbidity standards, followed by dilution (1:20 v/v) in the 7H9 broth. Each well was inoculated with 100 µL of this bacterial suspension. The plates were then incubated at 37 ± 1ºC for approximately 10 days. All the experiments were performed in triplicate.



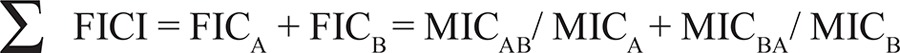



The data were analysed after calculating the fractional inhibitory concentration index (FICI) as follows:

Where MIC_AB_ equals the MIC of drug A in combination with drug B; MIC_A_ is the MIC of drug A alone; MIC_BA_ equals the MIC of drug B in combination with drug A and MIC_B_ is the MIC of drug B alone. The interaction is considered synergistic for FICI < 0.5; additive (0.5 ≤ FICI ≤ 1); indifferent (1 < FICI ≤ 4) and antagonistic (FICI > 4) (Agertt et al. 2013).

## RESULTS

*Physicochemical characterisation of liposomes* - UA-Lipo prepared with a 14:1 lipid:drug molar ratio (80 mM of lipids) exhibited a mean particle size of 146.46 ± 1.91 nm, PDI of 0.32 ± 0.01, surface charge of + 21.30 ± 0.51 mV and the drug efficiency ratio was practically 100% (99.56 ± 0.74%).

*Antimycobacterial activity* - *MIC* - The antimycobacterial activity of UA, UA-Lipo, as well as reference drugs against MDR-TB clinical isolates is shown in Table I. The susceptibility of each Mtb strain to UA and UA-Lipo were 31.25 µg/mL and 0.98 µg/mL, respectively, for all the strains tested while the MIC of INH and RIF varied. As expected, 0.5% methanol and empty liposomes did not exhibit any antimycobacterial activities.

**TABLE I:** Antimycobacterial activity of compounds tested against *Mycobacterium tuberculosis* clinical isolates

*M. tuberculosis* strains	MIC (µg/mL)			

	INH	RIF	UA	UA-Lipo
H37Rv	< 0.25	< 0.12	31.25	0.98
1412	≥ 16	4	31.25	0.98
1619	16	8	31.25	0.98
0729	≥ 16	≥ 8	31.25	0.98
1411	16	8	31.25	0.98
1409	4	≥ 8	31.25	0.98
1484	≥ 16	≥ 8	31.25	0.98

H37Rv: virulent strains; INH: isoniazid; MIC: minimum inhibitory concentration; RIF: rifampicin; UA: usnic acid; UA-Lipo: usnic acid encapsulated into liposomes.

*Checkerboard method* - The in vitro interactions were evaluated in two MDR-TB isolates (1619 and 1411) as these isolates presented the MIC values set for the reference and tested drugs. The interaction results of UA and UA-Lipo with INH and RIF against MDR-TB isolates are shown in Table II. The FICI of the combinations UA/INH and UA-Lipo/INH ranged from 1.30-2.5, exhibiting an indifferent effect. However, the combinations of UA/RIF and UA-Lipo/RIF exhibited a synergistic effect, resulting in a value below the threshold used to determine synergism (FICI < 0.5).

**TABLE II:** Effect of usnic acid or UA-into liposomes in combination with isoniazid or rifampicin against multidrug-resistant *Mycobacterium tuberculosis* clinical isolates

Combination	MDR-TB clinical isolates	MIC in combination (µg/mL)				FICI	Interaction

		UA	UA-Lipo	INH	RIF		
UA/INH	1619	31.25	-	4	-	1.30	Indifferent
	1411	62.5	-	16	-	2.50	Indifferent
UA-Lipo/INH	1619	-	0.98	16	-	2.00	Indifferent
	1411	-	62.5	4	-	2.25	Indifferent
UA/RIF	1619	7.81	-	-	0.5	0.31	Synergistic
	1411	7.81	-	-	1	0.38	Synergistic
UA-Lipo/RIF	1619	-	0.122	-	1	0.25	Synergistic
	1411	-	0.245	-	0.25	0.28	Synergistic

FICI: fractional inhibitory concentration index; INH: isoniazid; MDR-TB: multidrug-resistant *M. tuberculosis*; MIC: minimum inhibitory concentration; RIF: rifampicin; UA-Lipo: usnic acid encapsulated into liposomes.

## DISCUSSION

In this paper, conventional liposomes were selected as vehicles for UA because they are suitable carriers for antibiotics used in the treatment of intracellular pathogens, such as Mtb (Drulis-Kawa & Dorotkiewicz-Jach 2010).

Lira et al. (2009) reported a high encapsulation ratio of UA of approximately 96%, in their pioneer paper on liposomes containing UA (10:1 lipid:drug molar ratio, 42 mM of lipids). The present results indicate that the increase in the total lipid concentrations to 80 mM influenced the drug loading in liposomes (UA = 2 mg/mL), in comparison with our previous study (42 mM of lipids and UA = 1.5 mg/mL) (Lira et al. 2009).

Initially, the resistance of all the clinical isolates used in the present paper was thus confirmed, given that Ganihigama et al. (2015) reported MDR-TB isolates based on their resistance to INH and RIF (MIC values of 2- ≥ 8 µg/mL).

The antimycobacterial activity of UA is in agreement with those reported in the literature. Honda et al. (2010) evaluated the anti-tubercular activity of lichen derivatives against Mtb (H37Rv), including UA, with a MIC of 62.5 µg/mL. In the same year, Ramos and Silva determined the antimycobacterial activity of UA against resistant and susceptible Mtb clinical isolates. They found MIC values of 12.25 µg/mL for the standard sensitive strain H_37_Rv and 1.56-12.5 µg/mL for Mtb clinical isolates with monoresistance to INH, SM or RIF. Regarding the UA-Lipo, all isolates exhibited MIC values (0.98 µg/mL) more than 30-fold lower than UA alone. This phenomenon observed in MIC for all isolates may be explained in the sense of these strains have never come into contact with UA and UA-Lipo earlier, and thus have a certain degree of susceptibility and uniformity.

One possible explanation for the enhancement of antimicrobial activity owing the encapsulation of UA into lip- osomes may be attributed to electrostatic interactions between negatively-charged carboxyl groups of mycolic acids, which are the main components of the Mtb cell wall (Pi- nheiro et al. 2013), and positively-charged liposomal vesicles. In addition, liposomes and bacteria can interact directly by fusion processes, leading to the release of the encapsulated antibiotic within the bacteria (Sachetelli et al. 2000).

Other authors also have found that the encapsulation of antibiotics into liposomes enhances the antimycobacterial activity as compared with pure drugs. In 2009, Changsan et al. (2009) encapsulated RIF into liposomes using the thin film method. The MIC value of RIF encapsulated into liposomes against *Mycobacterium bovis* was 0.2 µM, whereas the MIC of RIF was higher (0.8 µM), thus indicating that liposomes were more efficient. Recently, Rojanarat et al. (2012) prepared proliposomes containing levofloxacin (LEV-proliposome) and its activity against *M. bovis* was assessed. The antimycobacterial activity of LEV-proliposomes was higher than LEV (MIC = 0.5 and 1 µg/mL, respectively). These in vitro results demonstrate that the encapsulation of drugs into liposomes may boost their antimycobacterial activity.

Yempala et al. (2013) synthesised novel dibenzofuran derivatives through molecular hybridisation and evaluated their antimycobacterial activity against Mtb H37Rv. Among the compounds analysed the lowest MIC value was 3.12 µg/mL. In comparison with our results, one may state that the encapsulation of UA into liposomes was three times more effective than the new UA derivatives.

The checkerboard method was used to verify the possible interaction effects of the combination of drugs on the antimicrobial activity of the compounds tested against Mtb. The drug combination regimens could lead to successful therapeutic schemes for TB treatment, considering MDR-TB isolates can rapidly develop resistance to new drugs, especially those redesigned from the existing scaffolds of the currently used anti-TB drugs (Ganihigama et al. 2015).

In synergism, the drugs involved can affect different targets in the microorganisms (Rastogi et al. 1998, Agertt et al. 2013). Thus the result obtained with the combinations of UA and UA-Lipo with RIF demonstrated an enhancement of their antimycobacterial activity, when compared with the effect of both drugs used separately.

Bapela et al. (2006) were pioneers in experiments combining natural products with antituberculous agents, such as INH and RIF. The combinations of INH or RIF with 7-methyljuglone, a natural product isolated from *Euclea natalensis*, reduced MIC values four fold and eightfold, respectively. Results showed a synergistic effect for both combinations against Mtb isolates.

In 2013, Rey-Jurado et al. (2013) evaluated the in vitro effect of combinations between antituberculous agents such as INH, RIF and EMB against drug-susceptible clinical isolates. The FICI values for all isolates were 1.5, showing indifferent effect. We can, therefore, say that our results of combinations between UA or UA-Lipo and RIF were more effective than the first-line drug combinations proposed by those authors.

Thus, UA-Lipo may be used as a dosage form to improve the antimycobacterial activity of RIF, a first-line drug for the treatment of infections caused by Mtb.
